# CD39/CD73-mediated immunosuppression and tumor aggressiveness in bladder cancer

**DOI:** 10.1007/s00262-026-04400-4

**Published:** 2026-04-22

**Authors:** Frederico Furriel, Paula Laranjeira, Margarida Pereira, Sandra Silva, Isabel Silva, Guilherme Fontinha, Vítor Sousa, Célia Gomes, Belmiro Parada, Artur Paiva

**Affiliations:** 1 Faculty of Medicine, University of Coimbra (FMUC), Coimbra, Portugal; 2 Unidade Local de Saúde Região de Leiria, Department of Urology, Hospital Santo André, Leiria, Portugal; 3 Department of Urology, CUF Coimbra Hospital, Coimbra, Portugal; 4 Flow Cytometry Unit, Clinical Pathology Department, Hospitais da Universidade de Coimbra, Unidade Local de Saúde de Coimbra, Coimbra, Portugal; 5 CIBB—Center for Innovative Biomedicine and Biotechnology, University of Coimbra, Coimbra, Portugal; 6 Faculty of Medicine (FMUC), Coimbra Institute for Clinical and Biomedical Research (iCBR), University of Coimbra, Coimbra, Portugal; 7 Clinical Academic Center of Coimbra (CACC), Coimbra, Portugal; 8 Unidade Local de Saúde de Coimbra, Department of Pathology, Hospitais da Universidade de Coimbra, Coimbra, Portugal; 9 Unidade Local de Saúde de Coimbra, Department of Urology and Renal Transplantation, Hospitais da Universidade de Coimbra, Coimbra, Portugal; 10 Ciências Biomédicas Laboratoriais, ESTESC-Coimbra Health School, Instituto Politécnico de Coimbra, Coimbra, Portugal

**Keywords:** Adenosinergic pathway, CD39, CD73, Immune profiling, Tumor immune evasion, Urinary bladder neoplasms

## Abstract

Urothelial bladder cancer (BCa) is marked by high recurrence and mortality, and the efficacy of PD-1/PD-L1 immunotherapy remains limited because of immune evasion. The adenosinergic pathway (AP), mediated by ectonucleotidases CD39 and CD73, is a key immunosuppressive mechanism, but its role in BCa remains unclear. We conducted an integrated immunophenotypic analysis of peripheral blood (PB) and the tumor microenvironment (TME) from 39 patients with BCa and 14 healthy controls using multicolor flow cytometry and immunohistochemistry. High-risk (HR) patients exhibited systemic immunosuppression, characterized by an elevated neutrophil-to-lymphocyte ratio and increased circulating regulatory T cells (Tregs), along with reduced cytotoxic γδ T cells and diminished Th1/Tc1 functional subtypes. In the TME, we observed reduced CD8^+^ T cell infiltration accompanied by increased Tregs, and phenotypes characterized by poor immune infiltration in the tumor core predominated across the cohort. In both PB and the TME, CD39 and CD73 expression on T cells strongly correlated with an immunosuppressive environment, marked by increased M2-like macrophages and decreased effector T cells. Circulating double-positive T cells (CD4^+^CD39^+^CD73^+^ and CD8^+^CD39^+^CD73^+^) mirrored the intratumoral T-cell composition, suggesting their potential as non-invasive biomarkers. Elevated frequencies of circulating single-positive CD8^+^CD39^+^ and CD8^+^CD73^+^ T-cells were significantly associated with higher pathological grade and distinguished high-grade tumors with moderate accuracy (AUC > 0.70). This study demonstrates that BCa is characterized by extensive AP-linked immunosuppression, and that specific ectonucleotidase-expressing circulating T-cell subsets may serve as non-invasive biomarkers for assessing tumor infiltration and grade.

**Supplementary Information:**

The online version contains supplementary material available at 10.1007/s00262-026-04400-4.

## Introduction

Urothelial bladder cancer (BCa) is the ninth most common malignancy worldwide, causing over 220,000 annual deaths [1]. Non-muscle invasive BCa (NMIBC) has a high recurrence rate, and its progression to muscle-invasive or metastatic disease significantly lowers the 5-year survival rate to below 50% and 21%, respectively [2, 3]. Furthermore, many patients are also ineligible for cisplatin-based treatment due to comorbidities [4].

Immune checkpoint inhibitors (ICIs) targeting the PD1/PD-L1 pathway have become a key treatment for metastatic BCa, particularly in cisplatin-ineligible patients. However, durable responses are achieved in only 20%–30% of patients, largely because of immune evasion mechanisms [5]. Additional agents, such as enfortumab-vedotin, have been successfully combined with ICIs, underscoring the importance of combination strategies to overcome resistance.

Immune evasion, a hallmark of cancer, has been increasingly recognized in the pathophysiology of BCa, and linked to recurrence, progression and treatment resistance [6]. Among the various immunosuppressive mechanisms, the adenosinergic pathway (AP) has emerged as an important contributor to cancer immune evasion [7]. Under physiological conditions, tissue damage or hypoxia triggers the release of adenosine triphosphate (ATP) into the extracellular space. ATP is subsequently converted to extracellular adenosine (ADO) through the sequential action of two key ectonucleotidases: CD39 (ectonucleotidase triphosphate diphosphate hydrolase), which hydrolyzes ATP to ADP and AMP, and CD73 (5ʹ-nucleotidase), converting AMP to ADO. ADO stimulates P1 purinergic receptors expressed on various immune and non-immune cells [8].

The AP promotes immune-mediated mechanisms supporting tumorigenesis [8], including: (1) suppression of T cell proliferation and function, along with reduced production of key effector molecules such as IFN-γ, TNF-α and perforin [9]; (2) stimulation of regulatory T cells (Tregs) [9] and regulatory B cells (Bregs) [10]; and (3) modulation of other immune cells, including antigen-presenting cells, NK cells, dendritic cells, macrophages [11] and endothelial cells [8]. The ability of extracellular adenosine (eADO) to act on high-affinity A2 receptors and inhibit immune cell function has been extensively studied across different cancers [8]. However, in BCa, the biological significance of the AP remains unclear and, in some reports, contradictory. For instance, increased CD39 expression has been linked both to advanced tumor stage and poorer survival [12] and to less aggressive pathological features [13]. Conversely, high CD73 expression in BCa is inversely correlated with tumor aggressiveness [14], in contrast to other cancers—such as ovarian, prostate, renal cell, rectal, breast, gastric, and colorectal—in which CD73 expression is associated with poorer survival [15, 16]. This discrepancy underscores the need for further investigation into the specific role of the AP in BCa biology.

In this study, we conducted an integrated immunophenotypic analysis of peripheral blood and tumor tissue from patients with BCa. Our goal was to elucidate the relationship between AP-related markers, immune cell profiles, and tumor characteristics, and to identify potential biomarkers of tumor aggressiveness.

## Materials and methods

### Patients

We enrolled 39 consecutive, treatment-naïve patients with pathology-confirmed urothelial BCa scheduled for transurethral resection of bladder tumor (TURBT) or radical cystectomy (RC) between May and June 2020, along with age- and sex-matched healthy controls for peripheral blood (PB) analysis. Tumors were staged according to the 8th edition of the TNM classification [17] and graded using the WHO 1973 (G1-3) and 2004/2016 (LG/HG) systems [18]. Two risk groups were defined: low-risk (LR; pT1 LG) and high-risk (HR; pT1 HG and pT ≥ 2). This binary stratification was chosen to compare tumors with low biological aggressiveness against those sharing a high propensity for progression, as recognized by EAU risk stratification guidelines [18], while ensuring robust group sizes for the extensive multiparametric analyses.

Preoperative PB samples were collected in EDTA tubes at the Unidade Local de Saúde de Coimbra (ULS-C) and the Unidade Local de Saúde da Região de Leiria (ULS-RL). During surgery, in addition to tumor tissue, macroscopically normal-appearing bladder mucosa adjacent to the tumor was collected to serve as a matched pairwise internal control (referred to hereafter and in figures as ‘normal tissue’ or ‘matched adjacent non-tumoral tissue’. Each tumor specimen was divided: one portion was fixed in 10% buffered formalin for pathology, and the other was preserved in PBS for flow cytometry. Age- and sex-matched individuals without known neoplastic or inflammatory disease were recruited as controls for PB. The demographic and clinical characteristics of patients and controls are summarized in Table 1.

**Table 1:** Main demographic and clinical features of the studied groups

	Patients (n = 39)	Controls (n = 14)	p
Age (mean ± SD)	76.4 ± 9.5	77.4 ± 10.7	NS
Male gender (%)	31 (79%)	12 (85%)	NS
Smoking status Non-smoker Past-smoker Active smoker	16 (41%) 14 (36%) 9 (23%)	6 (43%) 5 (36%) 3 (21%)	NS NS NS
Sample TURB Cystectomy	33 (85%) 6 (15%)	– –	– –
Tumor diameter (mean ± SD), mm	38.75 ± 3.12	–	–
pT stage pT1 pT ≥ 2	27(69%) 12 (31%)	– –	– –
Pathological Grade WHO 2004/2016 Low Grade High Grade WHO 1973 G1 G2 G3	22 (56%) 17 (44%) 5 (13%) 19 (49%) 15 (38%)	– – – – –	– – – – –
Risk Group* Low-risk High-risk	22 (56%) 17 (44%)	– –	– –

SD, standard deviation; NS, non-statistically significant; TURBT, transurethral resection of bladder tumor; pT, pathological T stage in TNM classification; WHO, World Health Organization; *Low risk low-grade pTa/1; *high-risk* high-grade pTa/1 or any pT ≥ 2

### Study approval

This study was approved by the Ethics Committees of the Faculty of Medicine, University of Coimbra (CE-151/2019), ULS-C (CHUC-137-19), and ULS-RL (CHL-CE-7/20). All participants provided written informed consent before enrollment.

### Pathology

Formalin-fixed, paraffin-embedded (FFPE) tissues were sectioned at 3 µm, with the first slide stained with hematoxylin and eosin (H&E) and subsequent slides used for immunohistochemistry (IHC). Automated IHC was performed on a Ventana Benchmark ULTRA platform using an OptiView DAB kit (Ventana Medical Systems, Inc.™, Tucson, USA). The monoclonal antibodies used are listed in Table 2. All slides were scanned at ×20 magnification (Aperio CS2, Leica) and analyzed using Aperio eSlide Manager (Leica Biosystems, Wetzlar, Germany).

**Table 2:** Monoclonal antibodies used for immunohistochemistry (IHC)

Target Protein	Clone/Type	Dilution	Manufacturer
PD-1	NAT105	1:50	Cell marque corporation
CD39	EPR20627	1:50	Abcam
CD73	Polyclonal	1:100	Abcam
Adenosine A2AR	EPR23731-50	1:25	Abcam
Adenosine A2BR	Polyclonal	1:500	Abcam
PD-L1	22C3	01:50	Agilent technologies
CD4	SP35	Ready to use	Ventana medical systems
CD8	C8/144B	1:100	Agilent technologies

A2AR, Adenosine A2A Receptor; A2BR, Adenosine A2B Receptor; CD, cluster of differentiation; PD-1, programmed death protein 1; PD-L1, Programmed death-ligand 1. *Commercial sources* Cell Marque Corporation, Rocklin, USA, Abcam, Cambridge, UK, Agilent Technologies™, Santa Clara, USA

A uropathologist (VS) recorded the diagnosis, grade, and growth pattern. IHC scoring for CD39, CD73, HIF1α, PD-1, and adenosine receptors included estimating the percentage and intensity (0–3 scale) of staining on tumor and immune cells. For CD4 and CD8, staining of tumor cells and the density (cells per high-power field [HPF]) in the tumor bulk and periphery were evaluated. PD-L1 was scored using standard metrics, including the combined positive score (CPS), immune cell score (IC), and tumor proportion score (TPS) [19].

Tumors were also classified into immune-inflamed (II), immune-excluded (IE), and immune-desert (ID) phenotypes based on the spatial distribution of CD8^+^ T cells [20]. Cutoffs, adapted from the BCa literature [21], were defined as the number of cells per HPF. Phenotypes were defined as follows: II (> 5 CD8^+^ T cells/HPF in the tumor core); IE (≤ 5 CD8^+^ T cells/HPF in the core and > 10 in the periphery); and ID (≤ 5 CD8^+^ T cells/HPF in the core and ≤ 10 in the periphery).

### Immunophenotypic study

Routine pre-operative peripheral blood complete blood counts (CBC) were performed by the hospital’s clinical pathology laboratory. The Neutrophil-to-Lymphocyte Ratio (NLR) was calculated by dividing the absolute neutrophil count by the absolute lymphocyte count obtained from these CBC results.

Immunophenotypic characterization of immune cells from peripheral blood (PB) and from tumoral and normal-appearing bladder mucosa adjacent to the tumor was performed using flow cytometry with a three-tube panel (Table 3).

**Table 3:** Monoclonal antibody panel for the identification and characterization of T cells and monocytes/macrophages

Tube		FITC	PE	PerCP-Cy5.5	PE-Cy7	APC	APC-H7	V450 BV421	BV510
1	Antibody marker	CD25	CD39	CD3	CD73	CD8	CD4	HLA-DR	CD127
	Clone	2A3	TU66	SK7	AD2	SK1	SK3	L243	HIL-7R-M21
	Commercial source	BD	BD Pharmingen	BD	BD Pharmingen	BD	BD	BD	BD
2	Antibody marker	TCRγδ	CD56	CD3	CCR4	CCR6	CD8	CD4	CCR5
	Clone	IMMU 510	MY31	SK7	L291H4	11A9	SK1	RPA-T4	2D7/CCR5
	Commercial source	Beckman Coulter	BD	BD	BioLegend	BD Pharmingen	BD	BD	BD
3	Antibody marker	HLA-DR	–	CD206	CD16	CD33	CD14	CD274	CD45
	Clone	L243		15–2	368	P67.6	MOP9	29E2A3	2D1
	Commercial source	BD		BioLegend	BD Pharmingen	BD	BD	BioLegend	BD

APC, allophycocyanin; APC-H7, allophycocyanin-hillite 7; BV, brilliant violet; CD, cluster of differentiation; CCR, C–C motif chemokine receptor; FITC, fluorescein isothiocyanate; PE, phycoerythrin; PE-Cy7, phycoerythrin–cyanine 7; PerCP-Cy5.5, peridinin chlorophyll protein-cyanine 5.5; TCR, T cell receptor. Commercial sources: BD (BD Biosciences), San Jose, CA, USA; BD Pharmingen, San Diego, CA, USA; Beckman Coulter, Miami, FL, USA; BioLegend, San Diego, CA, USA

Tumoral and peritumoral tissue was mechanically dissociated in phosphate-buffered saline (PBS; Gibco, Life Technologies, Carlsbad, CA), washed, and resuspended in 750 µL of PBS. The cell suspension was then distributed into three 75 × 12 mm flow cytometry tubes (250 µL per tube). In parallel, 100 μL of PB was transferred to three additional flow cytometry tubes.

Samples were immediately stained with the monoclonal antibodies (mAbs) listed in Table 3, followed by a 10 min incubation in the dark at room temperature. After a lyse-and-wash procedure, each sample was acquired on a FACSCanto II flow cytometer (BD Biosciences, San Jose, CA, USA) equipped with FACSDiva software (version 6.1.2, BD).

### Flow cytometry data acquisition and analysis

Data were analyzed using Infinicyt software (version 2.0.5; Cytognos SL, Salamanca, Spain). Two major subsets of CD3^+^ T cells were defined based on TCRγδ expression: γδ T cells (TCRγδ^+^) and αβ T cells (TCRγδ-). Among αβ T cells, subsets were defined based on CD4 and CD8 expression: CD4^+^CD8^−^, CD8^+^CD4^−^, CD4^+^CD8^+^, and CD4^−^CD8^−^ T cells. Tregs were identified within the CD4^+^ and CD8^+^ compartments based on a CD25^bright^ CD127^dim/negative^ phenotype. To preserve the in vivo native immunophenotype of the cells, we utilized surface chemokine receptors as established surrogate markers for T-cell functional polarization [22]. Each T cell subset was further divided according to expression of C–C motif chemokine receptor 6 (CCR6), in CCR6^+^ (T17-like cells), and CCR6^−^CCR5^+^ (T1-like cells), CCR6^−^CCR4^+^ (T2-like cells). The percentage of CCR6^+^ T cells co-expressing CCR5 (CCR6^+^CCR5^+^) or CCR4 (CCR6^+^CCR4^+^) was calculated, corresponding to T17 cells with plasticity toward T1- and T2-like phenotypes, respectively. The percentage of cells expressing CD39 and/or CD73 was evaluated in all T-cell subsets, and protein levels of CD39 and CD73 were measured as mean fluorescence intensity (MFI).

Monocytes/macrophages (CD45^+^, CD33^++^, and HLA-DR^+^) were identified as classical (CD14^+^CD16^−^), intermediate (CD14^+^CD16^++^) and nonclassical (CD14^low^/CD16^+^) and further subdivided into M1-like (CD206^−^) and M2-like (CD206^+^) populations. Neutrophils were identified by high side scatter (SSC) and forward scatter (FSC) properties, CD33 positivity, and lack of HLA-DR expression. Eosinophils were identified by the highest FSC and SSC among leukocytes and by their characteristic autofluorescence. Natural killer (NK) cells were defined phenotypically as CD3^−^/CD56^+^ cells. Finally, dendritic cells were identified by higher expression of CD33 and HLA-DR, together with lower expression of CD45 and lower SSC compared with monocytes. The main flow cytometry gating strategies and representative plots for relevant immune populations in PB and tumor samples are detailed in Supplementary Figs. 1 and 2, respectively.

### Statistical analysis

Statistical analyses were performed using SPSS (version 29; IBM, Chicago, USA) and GraphPad Prism (version 10; GraphPad LLC, Boston, USA). Data normality was assessed using the Shapiro–Wilk test. Depending on data distribution, two-group comparisons were performed using unpaired Student’s t tests or Mann–Whitney *U* tests. Multi-group comparisons were conducted using Kruskal–Wallis tests with Holm-Šidák correction. Fisher’s Exact Test was applied for categorical variables. Receiver operating characteristic (ROC) curve analysis was performed to evaluate biomarker performance. Statistical significance was set at *p* < 0.05.

## Results

### High-risk bladder cancer is characterized by systemic and local immunosuppression

**Fig. 1:**
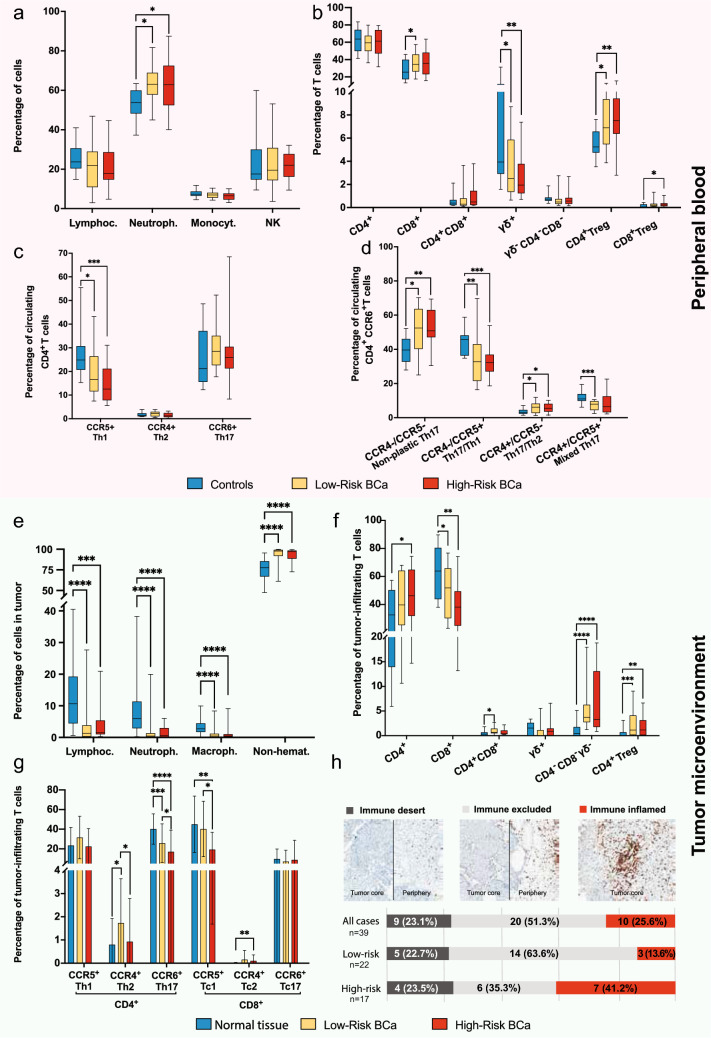
Immune populations in peripheral blood (PB) and tumor microenvironment (TME) in patients with BCa (n = 39), subdivided into Low-Risk (n = 22) and High-Risk cohorts (n = 17), compared to controls (n = 14) in the PB and normal tissue (n = 39) in the TME.**a** Distribution of the major immune cell populations in PB. **b** Distribution of T cell subsets in PB. **c** Percentage of Th17, Th1 and Th2 cells in PB. **d** Percentage of Th17 cells expressing the chemokine receptors CCR5 and/or CCR4 in PB. **e** Distribution of immune and non-hematopoietic (“Non-hemat.”, CD45^−^) cell populations in TME. **f** Distribution of T cell subsets in the TME. **g** Percentage of CD4^+^ and CD8^+^ T cells expressing the chemokine receptors CCR6 (Th17 and Tc17), CCR5 (Th1 and Tc1) and CCR4 (Th2 and Tc2) in the TME. **h** Immune Phenotype Distribution (Immune Desert, Immune-Excluded, Immune-Inflamed) based on CD8 + T Cell infiltration in the TME and its association with risk stratification in BCa. In panels **e**–**g**, the label ‘Normal tissue’ refers to matched, macroscopically normal adjacent non-tumoral bladder mucosa obtained from the same patients during surgery.Statistical significance was determined using the Mann–Whitney *U* test. *p < 0.05, **p ≤ 0.01, ***p ≤ 0.001, ****p ≤ 0.0001 when comparing the groups indicated in the figure

The main demographic and clinical characteristics of the studied groups are summarized in Table 1. The 39 patients with BCa and their matched controls were statistically comparable. Patients with BCa exhibited a significantly higher neutrophil-to-lymphocyte ratio in PB compared with controls (4.51 ± 4.46 vs. 2.28 ± 0.76, *p* = 0.036), whereas no significant differences were observed in monocyte or NK cell frequencies (Fig. 1a; Supplementary Table 1).

Compared with controls, PB from patients showed increased frequencies of both CD4^+^ and CD8^+^ regulatory T cells (Tregs) and decreased frequencies of γδ T cells, suggesting systemic immunosuppression and impaired antitumor surveillance, likely contributing to tumor immune evasion (Fig. 1b; Supplementary Table 1). Analysis of absolute cell counts confirmed these findings (data not shown).

Significant phenotypic changes were also observed in circulating T-cell populations among patients with BCa. Based on signature chemokine receptor expression, functional T-cell subsets were identified: Th17 and Tc17 cells (CCR6^+^), Th1 and Tc1 cells (CCR5^+^), Th17/Th1 and Tc17/Th1 (CCR5^+^CCR6^+^), and Th2/Tc2 cells (CCR4^+^). Compared with controls, patients with BCa, particularly those with HR tumors, exhibited reduced percentages of Th1 and Tc1 cells. This decline was evident across several T cell subpopulations, including CD8^+^ T cells, and reached statistical significance in CD4^+^ and CD4^+^CD8^+^ T cells (Fig. 1c, d; Supplementary Table 1). These findings suggest a shift away from type 1 (Th1/Tc1)-mediated antitumor immunity in patients with BCa, particularly in those with more aggressive disease. The decreased frequency of CCR5^+^ T cells reflect impaired cytotoxic function and a suppressed proinflammatory response, potentially contributing to tumor immune evasion.

Within the bladder, tumor tissue contained a lower proportion of total immune cells than adjacent normal tissue (Fig. 1e). Furthermore, tumor-infiltrating lymphocytes (TILs) displayed a distinct phenotype, with increased frequencies of CD4^+^ T cells, CD4^+^ Tregs, and triple-negative (CD4^−^ CD8^−^ γδ^−^) T cells, along with reduced levels of CD8^+^ T and NK cells (Fig. 1f; Supplementary Table 2). Consistent with PB findings, TILs from HR tumors displayed altered functional polarization, with fewer Tc1 and more Tc2 cells (Fig. 1g and Supplementary Table 2). Notably, even the adjacent non-tumoral tissue from HR patients differed from that of LR patients, with significantly higher frequencies of CD4^+^ Tregs (1.35 ± 1.74% vs. 0.13 ± 0.39%, *p* = 0.014 and lower frequencies of Tc1 cells (16.32 ± 3.29% vs. 41.72 ± 6.29%, *p* = 0.007) (Supplementary Fig. 3). These findings indicate a shift in the TME toward an immunosuppressive state, characterized by a loss of cytotoxic CD8^+^ T cells and enrichment of regulatory and dysfunctional T cell subsets. The reduction in Tc1 cells and expansion of Tc2 cells suggest a suppression of type 1 immune responses within tumors, particularly in high-risk aggressive disease, likely contributing to tumor progression and impaired immune surveillance.

Using predefined criteria, BCa samples were categorized according to CD8^+^ T cell infiltration in both the tumor core and periphery. Of the 39 samples analyzed, 10 (25.6%) were classified as II, 20 (51.3%) as IE, and 9 (23.1%) as ID. Immune phenotype distribution varied with disease risk: only 13.6% of low-risk cases were classified as II, compared to 41.2% in high-risk cases (Fig. 1h; Supplementary Table 3). Additionally, while faint, likely artifactual PD-1 staining was observed on some tumor cells (universally score 1 intensity), robust membrane PD-1 expression (score 3 intensity) was strictly localized to the immune infiltrate. The percentage of these robustly PD-1^+^ immune cells, indicative of functional exhaustion, was significantly (*p* = 0.04) higher in II cases (41.0 ± 12.9%), compared to IE cases (30.8 ± 15.8%).

### CD39/CD73 expression correlates with the immunosuppressive landscape and the spatial architecture of the TME

We next investigated whether the previously observed alterations in immune cell profiles were associated with expression of the ectonucleotidases CD39 and CD73 in both circulating and tumor-infiltrating T cells.

**Fig. 2:**
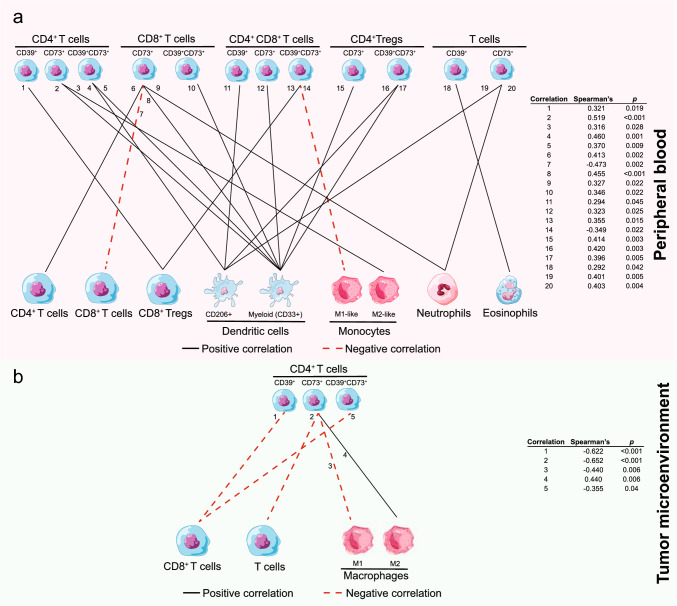
Correlation network of CD39 and/or CD73 expression on T cells subsets with immune populations in peripheral blood (**a**) and tumor microenvironment (**b**) (n = 39). Black solid lines represent positive correlations, and red dashed lines represent negative correlations. Statistical significance (*p*) and correlation coefficients (ρ) were calculated using Spearman’s rank correlation

In PB, the frequencies of distinct T cell subsets expressing CD39, CD73, or both, exhibited significant correlations with circulating immune populations. Specifically, we observed strong positive correlations between CD39^+^, CD73^+^ or CD39^+^CD73^+^ T cell subsets (including CD4^+^, CD8^+^, and CD4^+^ Tregs) and immunosuppressive populations such as CD8^+^ Tregs, M2-like monocytes, myeloid dendritic cells, with the strongest association found between CD4^+^CD73^+^ T cells and myeloid (CD33^+^) dendritic cells (ρ = 0.519, *p* < 0.001). Conversely, significant inverse correlations were observed with key antitumor populations: CD8^+^CD73^+^ T cells negatively correlated with overall CD8^+^ T cells (ρ = − 0.473, *p* = 0.002), while double-positive CD4^+^CD8^+^CD39^+^CD73^+^ T cells negatively correlated with M1-like monocytes (ρ = − 0.349, *p* = 0.022) (Fig. 2a).

In the TME, ectonucleotidase expression also correlated with immunosuppressive features. Specifically, CD39^+^ CD4^+^ T cells inversely correlated with CD8^+^ T cell infiltration, while CD73^+^ CD4^+^ T cells were inversely correlated with γδ T cell infiltration (Fig. 2b). Accordingly, IHC showed increased expression of A2AR and A2BR in immune cells infiltrating HR tumors (Supplementary Table 3). Furthermore, CD73 expression on CD4^+^ T cells correlated positively with CD206^+^ M2-phenotype macrophages (Spearman’s ρ = 0.440. 95% CI 0.130 to 0.671, *p* = 0.006). We also evaluated correlations between the expression of AP-related markers in tumor cells and the immune landscape of the TME. Two significant correlations were observed: a positive correlation between the frequency of CD39^+^ tumor cells and increased tumor infiltration of CD4^+^ Tregs (Spearman’s ρ = 0.440, 95% CI 0.135 to 0.668, *p* = 0.006), and an inverse correlation between the proportion of A2BR^+^ tumor cells and CD8^+^ T cells infiltration (Spearman’s ρ = − 0.345, 95% CI − 0.602 to − 0.023, *p* = 0.037). This suggests a potential role for A2B signaling in the exclusion or dysfunction of cytotoxic T cells within the TME.

**Fig. 3:**
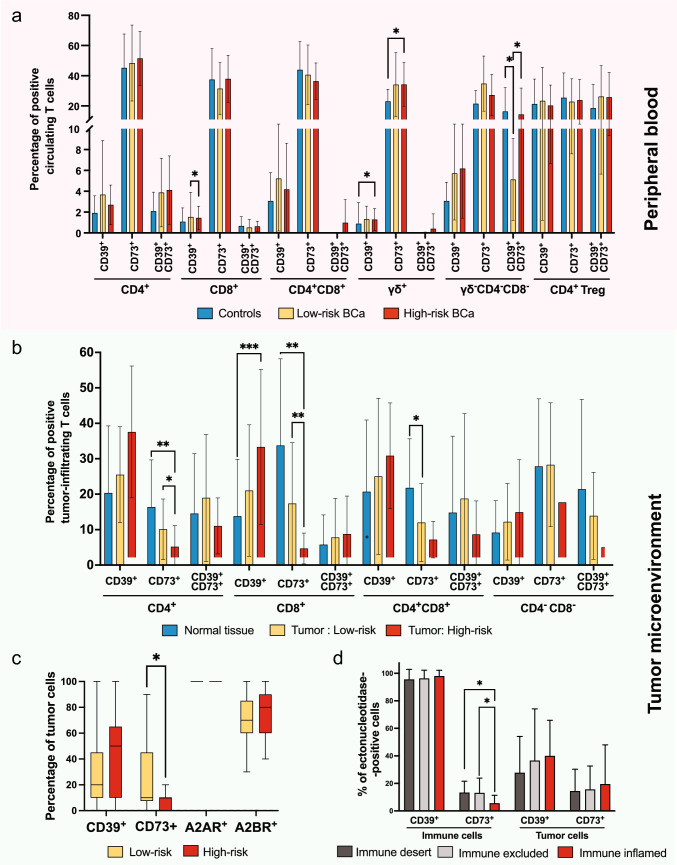
Ectonucleotidases expression in T cells and tumor cells from patients with BCa (n = 39), stratified by risk (Low-risk n = 22; High-risk n = 17) and immune phenotype (Immune-desert n = 9; Immune-excluded n = 20; Immune-inflamed n = 10),compared to controls in the PB (n = 14) and normal tissue(n = 39). **a** Percentage of CD39^+^, CD73^+^ and CD39^+^CD73^+^ cells within the various T cells subsets in PB. **b** Percentage of CD39^+^, CD73^+^ and CD39^+^CD73^+^ cells within the various T cells subsets in the TME. The label ‘Normal tissue’ denotes matched adjacent non-tumoral bladder mucosa obtained from the same patients **c** Percentage of tumor cells expressing CD39, CD73, Adenosine A2A receptor (A2AR) or Adenosine A2B receptor (A2BR). **d** Percentage of immune and tumor cells expressing CD39 or CD73, according to the immune phenotype (Immune Desert, Immune-Excluded, Immune-Inflamed). Statistical significance was determined using the Mann–Whitney *U* test. *p < 0.05, when comparing the groups indicated in the figure

Ectonucleotidases levels in PB were generally similar between healthy controls and patients with BCa. However, γδ T cells from patients with BCa, particularly those with HR disease, showed significantly higher levels of both CD39 and CD73 than controls. No significant differences were observed in PB between LR and HR groups (Fig. 3a). Across most circulating T cell subsets, CD73 positivity predominated over CD39. These findings suggest that although overall ectonucleotidase expression in blood remains relatively stable, specific subsets such as γδ T cells, may contribute to an immunosuppressive phenotype in more aggressive BCa. Higher CD73 expression across circulating T cells suggests a potential role in shaping the systemic immune response in patients with BCa.

In contrast, within the TME, CD39 and CD73 expression were more balanced among T cell subsets, although CD39 predominated slightly on CD4^+^, CD8^+^, and CD4^+^CD8^+^ T cells. Conversely, the frequencies of CD4^+^CD73^+^ and CD8^+^CD73^+^ T cells were reduced in tumor tissue. These alterations were particularly evident in HR tumors, which showed substantial expansion of CD39^+^ T cells within both CD4^+^ and CD8^+^ subsets, along with a marked reduction in CD73 expression compared with LR tumors (Fig. 3b). Likewise, the proportion of tumor cells expressing CD73 was significantly lower in HR tumors than in LR cases (Fig. 3c).

When stratifying ectonucleotidases positivity by immune phenotype, we observed that although II tumors exhibited a higher density of immune cells, their CD73 positivity (5.6 ± 5.8%) is significantly reduced compared to ID (13.3 ± 8.3% *p* = 0.013) and IE phenotypes (13.1 ± 10.8%, *p* = 0.027) (Fig. 3d; Supplementary Fig. 4). These differences were not site-specific within the tumor samples, affecting both the core and periphery.

To fully contextualize these spatial distributions within our patient cohort, we mapped the immune phenotypes and corresponding CD73 expression patterns across the clinical risk groups. As outlined in our comprehensive study workflow (Supplementary Fig. 5), this stratification reveals that the marked reduction of CD73^+^ immune cells in the II phenotype occurs fundamentally in the high-risk cases. This indicates that the transition to an immune-inflamed state in more aggressive tumors involves a specific loss of CD73 expression on infiltrating immune cells.

### Circulating CD39^+^/CD73^+^ T cells serve as non-invasive biomarkers for tumor immune infiltration and grade

**Fig. 4:**
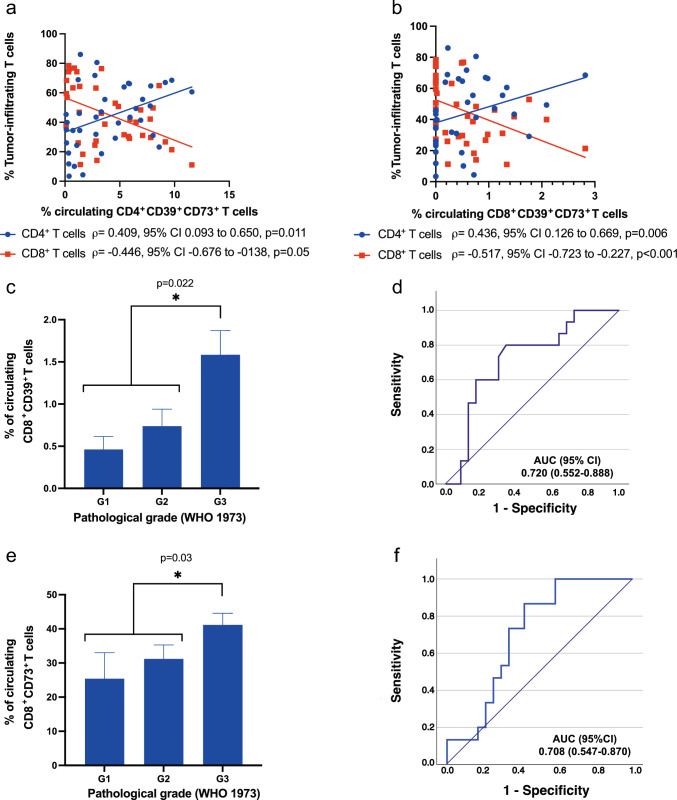
Peripheral blood AP-based biomarkers associated with immune tumor infiltration and pathological grade. **a** Scatter plot correlating the percentage of circulating CD4^+^CD39^+^CD73^+^ T cells with the percentage of tumor-infiltrating CD4^+^ (blue circles; positive correlation) and CD8 + (red squares; negative correlation) T cells. **b** Scatter plot correlating the percentage of circulating CD8^+^CD39^+^CD73^+^ T cells with the percentage of tumor-infiltrating CD4^+^ (blue circles; positive correlation) and CD8 + (red squares; negative correlation) T cells. For (A) and (B), lines of best fit are shown; statistical significance was determined using Spearman’s rank correlation test and the correlation coefficient (ρ), 95% confidence intervals (CI), and p-values are indicated. **c** Percentage distribution of circulating CD8^+^CD39^+^ T cells in PB across WHO 1973 pathological grades G1 (n = 5), G2 (n = 19), and G3 (n = 15). Bars represent mean ± standard error of the mean (SEM). **d** Receiver operating characteristic (ROC) curve analysis using the percentage of circulating CD8^+^CD39^+^ T cells to predict pathological grade G3. (E) Percentage distribution of circulating CD8^+^CD73^+^ T cells in PB across WHO 1973 pathological grades G1 (n = 5), G2 (n = 19), and G3 (n = 15). Bars represent mean ± SEM (F) ROC curve analysis using the percentage of circulating CD8^+^CD73^+^ T cells to predict pathological grade G3. AUC, Area Under the Curve; CI, confidence interval. Statistical significance for group comparisons in (**c**) and (**e**) was determined using the Mann–Whitney *U* test and the significance of the AUC in the ROC analyses (**d**) and (**f**) was calculated using the Wilcoxon method

We next investigated whether peripheral blood markers correlated with TME characteristics. Circulating CD4^+^CD39^+^CD73^+^ and CD8^+^CD39^+^CD73^+^ correlate positively with tumor-infiltrating CD4^+^ T cells and negatively with tumor-infiltrating CD8^+^ T cells (Fig. 4a, b). These findings suggest that ectonucleotidase expression in peripheral T cells reflects the immune composition in the TME, specifically an immune profile skewed toward immunoregulatory CD4^+^ T cell dominance and reduced cytotoxic CD8^+^ T cell infiltration. Receiver operating characteristic (ROC) analysis supported this relationship, indicating that increased levels (≥ median) of circulating CD4^+^CD39^+^CD73^+^ and CD8^+^CD39^+^CD73^+^ T cells could predict high CD4^+^ T cell infiltration within tumors with moderate diagnostic accuracy (AUC = 0.712, 95% CI: 0.544–0.880 for CD4^+^ cells; AUC = 0.719, 95% CI: 0.553–0.885 for CD8^+^ cells). Furthermore, while these relevant associations were identified between the PB and the TME, parallel correlation analyses comparing circulating AP features with immune populations in the matched adjacent non-tumoral tissue yielded no significant correlations.

We also identified a significant association between ectonucleotidase expression on circulating T cells and pathological tumor grade. Specifically, the mean percentage of circulating CD8^+^CD39^+^ T cells was significantly higher (*p* = 0.022) in patients with WHO 1973 grade 3 tumors (1.58 ± 1.11%) than those with Grade 1 (0.46 ± 0.34%) or Grade 2 tumors (0.73 ± 0.81%) (Fig. 4c). ROC analysis demonstrated that a peripheral CD8^+^CD39^+^ T cell frequency above 0.8% distinguished Grade 3 tumors with 80.1% sensitivity and 65.2% specificity (AUC = 0.720, 95% CI: 0.552–0.888; Fig. 4d). Similarly, the percentage of circulating CD8^+^CD73^+^ T cell was significantly higher (*p* = 0.03) in patients with Grade 3 tumors (41.15 ± 13.18%) than in those with Grade 1 or 2 tumors (25.40 ± 17.01% and 31.20 ± 17.68%, respectively) (Fig. 4e). A threshold of 30% for CD8^+^CD73^+^ T cells predicted Grade 3 tumors with 86.7% sensitivity and 58.3% specificity (AUC = 0.708, 95% CI: 0.547–0.870; Fig. 4f).

## Discussion

Immune evasion significantly impairs host defense against cancer cells and contributes to the limited effectiveness of PD-1/PD-L1 immunotherapy currently used to treat urothelial carcinoma. The AP has gained attention for its immunoregulatory role in various cancers [23]: however, its role in BCa is not yet fully characterized. Based on our recent preclinical evidence showing that co-targeting CD73 along with PD-1/PD-L1 blockade significantly enhances anti-tumor efficacy [24], we sought to evaluate the adenosinergic landscape in this human cohort. Our findings in patients with HR BCa reveal a systemic shift toward immunosuppression, evidenced by increased NLR, increased circulating Tregs, decreased cytotoxic γδ T cells, and reduced Th1/Tc1 cells. While elevated NLR is a recognized marker of systemic inflammation, it has also been associated with advanced tumor stages and grades, as well as poorer survival outcomes in various malignancies, including BCa [25]. The observed enrichment of circulating Tregs further supports their established role in suppressing antitumor immunity [26].

Notably, the marked reduction in circulating γδ T cells, particularly in high-risk patients, suggests impaired anti-tumor immunity, consistent with findings in other malignancies, where decreased γδ T cell levels in the PB predict worse prognosis. For instance, reduced circulating γδ T cells have been associated with aggressive follicular thyroid cancer [27] and with increased recurrence risk in renal cell carcinoma after surgery [28]. Likewise, the reduced frequency of CCR5^+^ CD4^+^ Th1 cells—key mediators of IFN-γ driven cellular immunity—reinforces an impaired peripheral cytotoxic immune profile in patients with BCa.

Within the TME, we also observed significant changes indicative of immune dysfunction. Beyond a general “immune dilution” from tumor proliferation, the TIL compartment showed a sharp reduction in the CD8^+^ T cell fraction, consistent with previous studies [12]. This reflects the fact that phenotypes characterized by an absence of cytotoxic T cells in the tumor core (ID and IE), when considered collectively, constitute the majority of cases (74.4%), a pattern associated with resistance to PD1/PD-L1 blockade. Even within the HR group, where the immune-inflamed phenotype was more frequent (41.2%), the combined non-inflamed phenotypes (ID and IE) still accounted for the majority (58.8%) of the tumors. [6]. This reduction was accompanied by an increase in Tregs—linked to worse prognosis in BCa [29] and by increased PD-1 positivity on immune cells (with mild tumor cell staining considered as artifactual due to cross-reactivity with nuclear antigens of dying cells [30]), particularly in inflamed tumors. The latter suggests functional exhaustion and aligns with previous studies correlating PD-1 positivity with unfavorable outcomes in urothelial cancer [31]. Critically, this impairment extended beyond the TME, as adjacent non-tumoral tissue from HR patients also showed increased Tregs and reduced Tc1 cells. This indicates that immunosuppression and impaired cytotoxic responses affect the entire organ, potentially predisposing it to malignant transformation [32].

Central to these immunological changes are the AP-associated ectonucleotidases CD39 and CD73, which sequentially hydrolyze extracellular ATP into immunosuppressive adenosine. Mechanistically, tumor hypoxia—evidenced by diffuse HIF-1α expression in our cohort—upregulates CD39 and CD73 while triggering the release of extracellular ATP. CD39^+^CD73^+^ T cells rapidly scavenge this ATP, converting it into eADO. The eADO then exerts potent autocrine and paracrine suppressive effects by binding to A2A and A2B receptors, which were highly expressed on infiltrating immune cells in our HR cohort. This receptor engagement elevates intracellular cAMP, directly inhibiting T-cell receptor signaling and effector cytokine production while stabilizing local regulatory T cells and M2-like macrophages [8].

In PB, the frequencies of CD39^+^, CD73^+^, and double positive (CD39^+^/CD73^+^) cells across various T cell subsets (CD4^+^, CD8^+^, CD4^+^CD8^+^, CD4^+^ Tregs and γδ) correlated positively with circulating immunosuppressive subsets including CD8^+^ Tregs [33], myeloid dendritic cells [34] and M2-like (CD206^+^) monocytes [35], while correlating inversely with effector CD8^+^ T cells [36] and M1-like (CD206^−^) monocytes [37].

Our observation that CD39 expression on CD4^+^ T cells within the TME negatively correlated with cytotoxic CD8^+^ T-cell infiltration aligns with its immunosuppressive role. This is supported by preclinical models showing that CD39 inhibition enhances CD8^+^T-cell function [38] and by clinical data linking circulating CD39^+^ CD8^+^ T cells to poor prognosis in other cancers [39]. The discrepant patterns of CD39 expression reported by Zhu et al. [12], in which it was associated with both low-risk features on T-cells and advanced stage in bulk tissue, highlight the need for cell-specific analysis, as performed here.

Likewise, the frequency of CD73^+^ CD4^+^ T cells in the TME inversely correlated with infiltration of cytotoxic γδ T cells, a population whose reduction is linked to poorer clinical outcomes in BCa [40]. Furthermore, these CD73-expressing CD4^+^ T cells were associated with a shift in macrophage polarization, correlating negatively with proinflammatory M1 macrophages and a positive one with immunosuppressive M2 macrophages [41]. Collectively, these findings suggest that, similar to CD39, the expression of CD73 on tumor-infiltrating CD4^+^ T cells is indicative of an immunosuppressive and protumorigenic state in the TME.

Our findings reveal complex, cell type–specific roles for CD39 and CD73 in BCa. We provide evidence that increased CD39 expression on T cells within the TME of HR tumors is a hallmark of an aggressive, immunosuppressive phenotype, consistent with its role in generating adenosine and suppressing cytotoxic responses. Conversely, we observed reduced CD73 expression on both T cells and tumor cells in these same HR cases. This contrasts with our correlation data showing CD73 associated with protumor features and with findings in other cancers. This apparent paradox may reflect a context-dependent role of CD73. For instance, Koivisto et al. [42] reported an inverse correlation between tumor aggressiveness and CD73 expression in tumor cells, although this association was not observed in T lymphocytes. Differences in methodological approaches, like cell-specific protein analysis versus bulk tumor mRNA measurement, may contribute to the discrepancies described above, highlighting the importance of evaluating the AP markers within specific cellular and spatial contexts of the TME. Additionally, CD73 downregulation may result from tumor cell adaptation or immune editing as tumors evolve toward more aggressive states. This dynamic immune editing is particularly evident when analyzing CD73 expression across different spatial immune phenotypes. In ID and IE tumors, the significantly higher CD73 expression observed on immune cells impairs proliferation and/or restricts cytotoxic cells to the periphery. In contrast, II tumors—which are predominantly found in HR cases—exhibit significantly reduced CD73 on immune cells. This reduction likely permits T-cell infiltration. However, these tumors still evade immune destruction by shifting from CD73-driven spatial exclusion to PD-1-mediated functional exhaustion, as evidenced by significantly higher PD-1 positivity on immune cells in II compared to IE cases. Although our cross-sectional design precludes evaluating treatment outcomes, this barrier-forming role of CD73 in non-inflamed tumors could theoretically impede therapies requiring robust immune infiltration, such as Bacillus Calmette-Guérin (BCG). Future longitudinal studies are needed to clarify whether evaluating CD73 in TURBT specimens can predict BCG response.

Clinically, the significant correlation between circulating CD39^+^CD73^+^ T cells, tumor lymphocytic infiltration, and pathological grade, suggests these cells could serve as non-invasive biomarkers of tumor immune status and aggressiveness. Although they represent a small fraction of circulating cells, a one- to threefold increase of both CD4^+^CD39^+^CD73^+^ and CD8^+^CD39^+^CD73^+^ was associated with higher CD4^+^ and lower CD8^+^ T cells levels within the tumor. While infiltrating CD4^+^ and CD8^+^ T cells often correlate positively in inflamed tumors, their inverse relationship here reflects a skewed CD4^+^ compartment, enriched for immunosuppressive Tregs (~ 20% vs. < 1% in normal tissue). Additionally, percentages of circulating CD8^+^CD39^+^ and CD8^+^CD73^+^ T cells were increased in high pathological grade tumors and demonstrated moderate ability to predict tumor grade. These findings highlight the potential of circulating CD39^+^/CD73^+^ T cells as biomarkers of tumor immune infiltration and grade, offering a non-invasive tool for patient stratification and treatment decisions, such as prioritization for surgery and prediction of response to checkpoint inhibitors [43]. Crucially, the fact that these robust correlations were found exclusively between the PB and the tumor—and not with the matched adjacent non-tumoral tissue—further reinforces their potential clinical validity. This also demonstrates that the systemic adenosinergic alterations observed in the circulation are driven by the TME rather than baseline bladder homeostasis. Furthermore, unlike invasive tissue biopsies that are subject to spatial heterogeneity, or emerging NGS-based liquid biopsies (e.g., ctDNA) that remain expensive with prolonged turnaround times, peripheral blood flow cytometry is a rapid, highly cost-effective technique already established in standard hospital laboratories. While this association is novel in BCa, increased levels of circulating CD8^+^CD39^+^ and CD8^+^CD73^+^ have been associated with poorer survival outcomes in cervical cancer [39] and melanoma [44], respectively. In head and neck squamous cell carcinoma, increased percentages of circulating CD73^+^ myeloid-derived suppressor cells have also been linked to advanced clinical stages [45]. These findings suggest that ectonucleotidase-based peripheral biomarkers could be integrated with other emerging tools such as circulating tumor DNA and cells, soluble factors (cytokines, chemokines, growth factors) or extracellular vesicles [46] to enhance diagnostic accuracy and prognostic assessment in BCa.

Despite these promising findings, this study has several limitations. First, our binary risk stratification grouped pT1 high-grade NMIBC and MIBC (pT ≥ 2) into a single high-risk cohort. While both share aggressive biological behavior and a high risk of progression, they are clinically distinct entities and combining them may obscure subtle differences in their respective immune architectures and AP activity. Furthermore, the small sample size and cross-sectional design limit generalizability, and larger prospective studies with long-term follow-up are needed to confirm associations with clinical outcomes, treatment response, and survival. Although we observed strong correlations, the observational nature of the study precludes conclusions about causality between AP activity and immune modulation in BCa. Incorporating comprehensive genetic and molecular analyses in future research will be critical for understanding how these factors shape the immune and adenosinergic landscape of BCa. Furthermore, while our exploratory analysis prioritized T cells as the primary anti-tumor effectors, future studies utilizing dedicated panels should explicitly evaluate the adenosinergic landscape across other populations, such as myeloid-derived suppressor cells (MDSCs). Nevertheless, our findings support further research into therapeutic strategies targeting the AP, such as CD39/CD73 inhibitors or A2A/A2B receptor antagonists, which are currently under clinical evaluation in other malignancies [47]. In the current rapidly evolving treatment landscape of BCa, where novel combinations of immune checkpoint inhibitors and antibody–drug conjugates (e.g., enfortumab vedotin) are becoming standard of care, understanding overlapping mechanisms of immune evasion is critical. Targeting the AP may offer a synergistic approach to overcome resistance in patients who do not achieve durable responses to these modern regimens.

### Conclusion

In summary, BCa is characterized by significant alterations in the immune profile, particularly in CD39 and CD73 expression, which contribute to significant immunosuppression in both PB and the TME. These patterns correlate with disease aggressiveness and show promise as non-invasive biomarkers. Further research is warranted to clarify the role of the AP and to validate its dual potential as a therapeutic target and prognostic tool in BCa.

## Supplementary Information

Below is the link to the electronic supplementary material.

Supplementary file1 (PDF 3018 KB)

Supplementary file2 (PDF 2216 KB)

Supplementary file3 (PDF 252 KB)

Supplementary file4 (PDF 3973 KB)

Supplementary file5 (PDF 393 KB)

Supplementary file6 (DOCX 33 KB)

Supplementary file7 (DOCX 22 KB)

Supplementary file8 (DOCX 26 KB)

## Data Availability

Due to legal and ethical restrictions, the clinical data used in this study cannot be shared publicly, as the patients did not provide consent. However, anonymized data relevant to the study findings are available from the corresponding author upon reasonable request.
