# Supplementary figures and images for: CD39/CD73-mediated immunosuppression and tumor aggressiveness in bladder cancer
Source: Cancer Immunol Immunother. 2026 Apr 22;75(5):154. doi: 10.1007/s00262-026-04400-4 (PMC13103164; doi:10.1007/s00262-026-04400-4)

Peripheral Blood

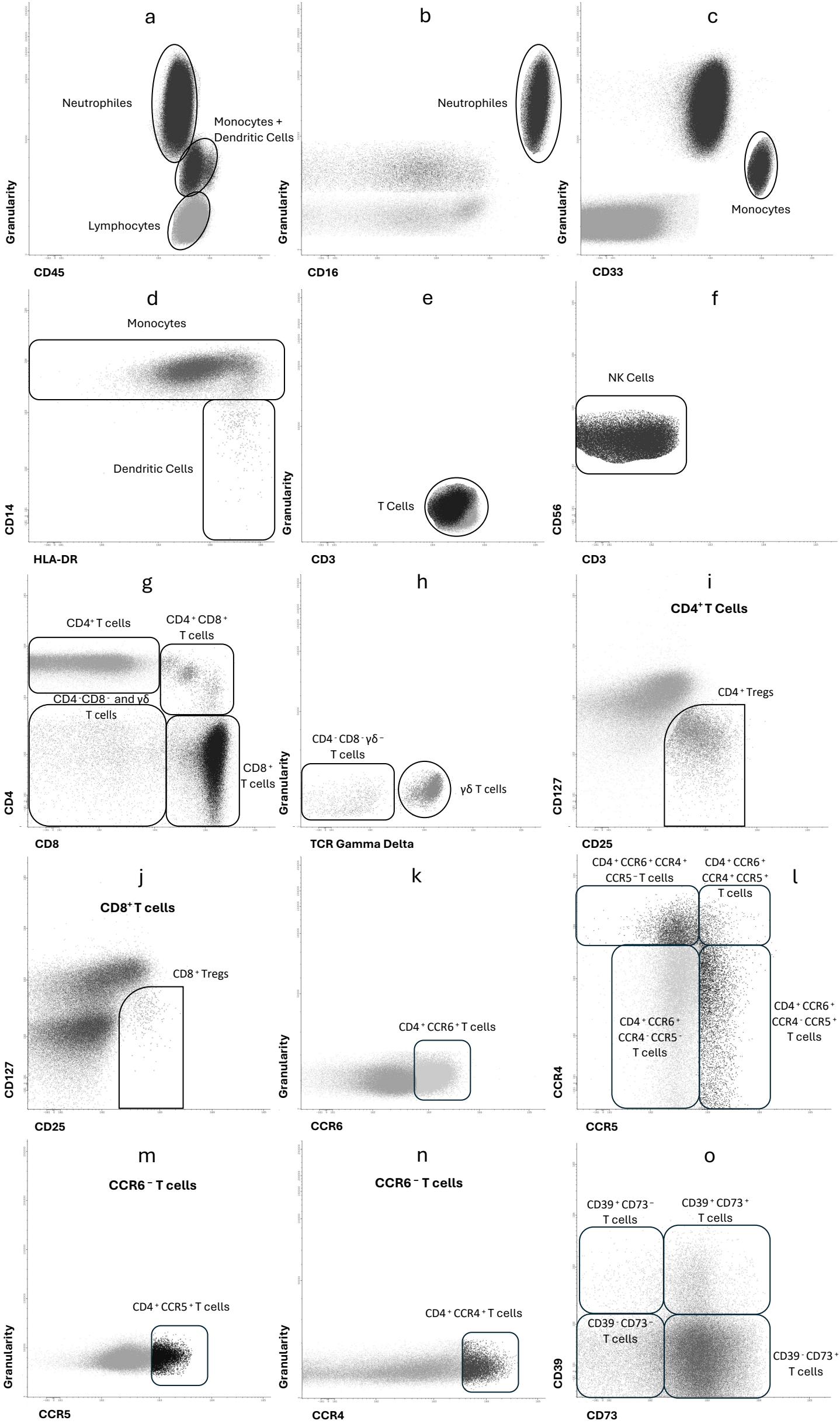

Supplement: Supplementary file 1 — Supplementary file1 (PDF 3018 KB) [file 262_2026_4400_MOESM1_ESM.pdf]

Tumor Microenvironment

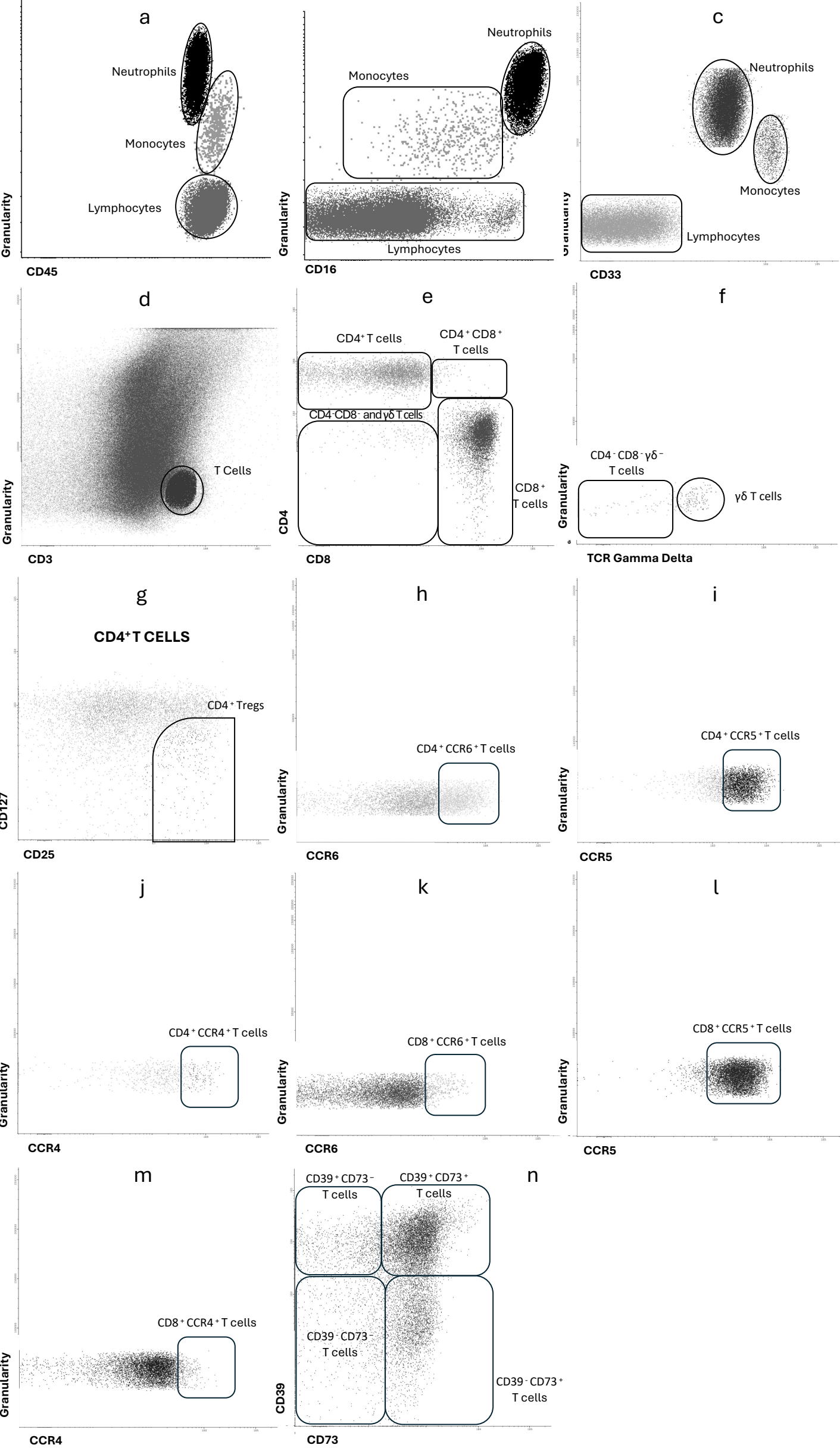

Supplement: Supplementary file 2 — Supplementary file2 (PDF 2216 KB) [file 262_2026_4400_MOESM2_ESM.pdf]
