# Supplementary material for: CD39/CD73-mediated immunosuppression and tumor aggressiveness in bladder cancer
Source: Cancer Immunol Immunother. 2026 Apr 22;75(5):154. doi: 10.1007/s00262-026-04400-4 (PMC13103164; doi:10.1007/s00262-026-04400-4)
Supplement: Supplementary file 3 — Supplementary file3 (PDF 252 KB) [file 262_2026_4400_MOESM3_ESM.pdf]

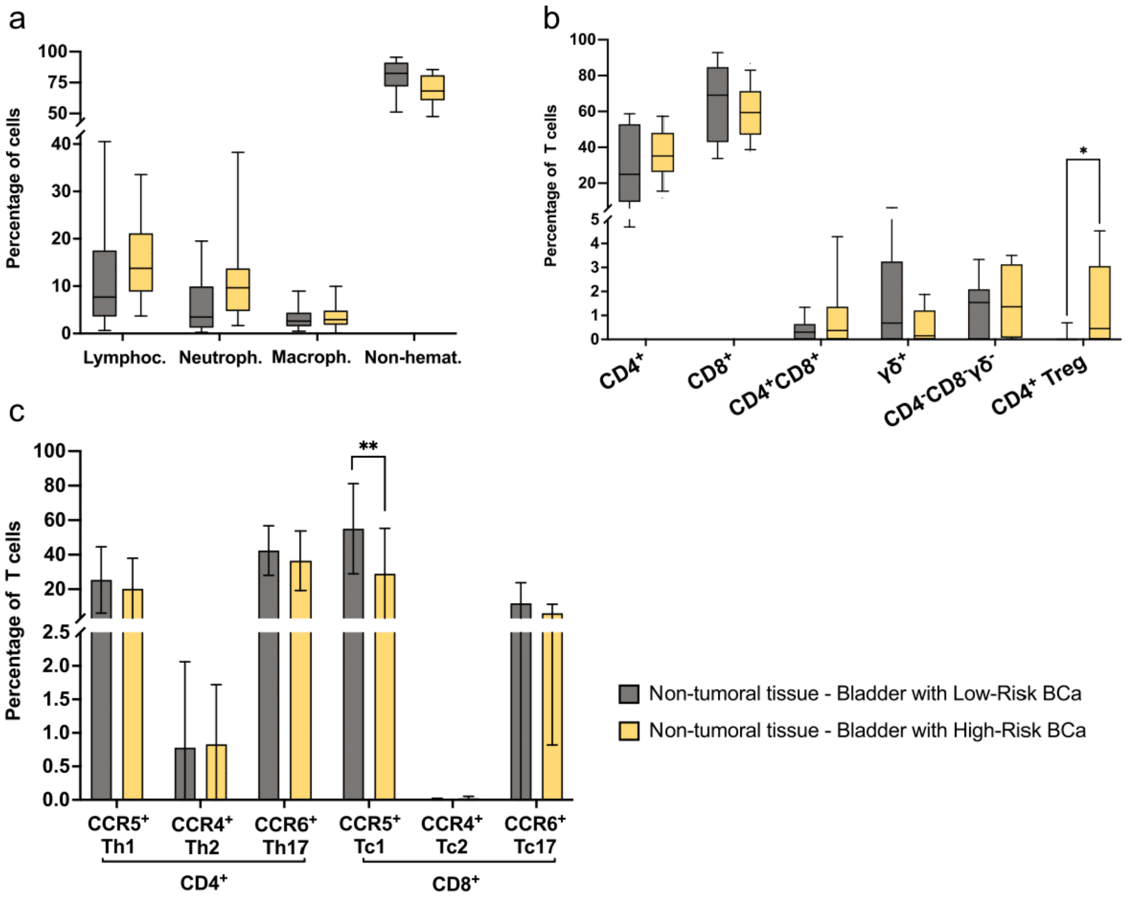

Supplementary Figure 3 Characterization of the immune environment in non-tumoral bladder tissue from patients with low-risk (LR) (n=22) versus high-risk (HR) (n=17) BCa. (A) Distribution of immune and non-hematopoietic cell populations in non-tumoral tissue. (B) Distribution of T cell subsets in non-tumoral tissue. (C) Percentage of CD4+ and CD8+ T cells expressing the chemokine receptors CCR6 (Th17 and Tc17), CCR5 (Th1 and Tc1) and CCR4 (Th2 and Tc2) in non-tumoral tissue
