# Supplementary material for: CD39/CD73-mediated immunosuppression and tumor aggressiveness in bladder cancer
Source: Cancer Immunol Immunother. 2026 Apr 22;75(5):154. doi: 10.1007/s00262-026-04400-4 (PMC13103164; doi:10.1007/s00262-026-04400-4)
Supplement: Supplementary file 4 — Supplementary file4 (PDF 3973 KB) [file 262_2026_4400_MOESM4_ESM.pdf]

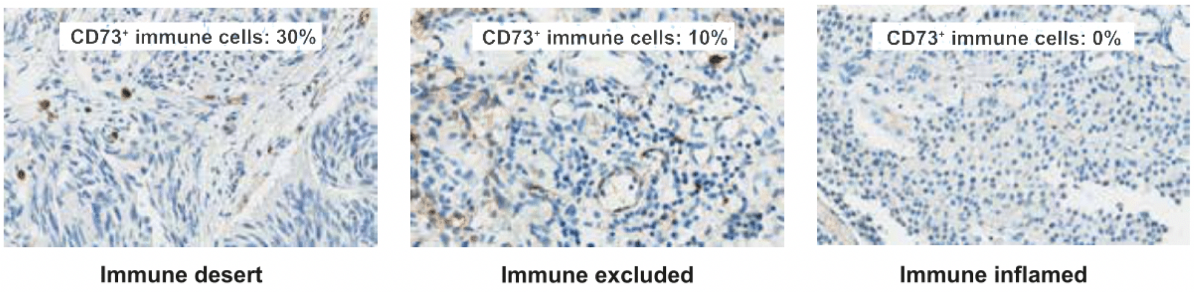


**Supplementary Figure 4** Representative images of CD73 expression in the tumor microenvironment across different immune phenotypes. Immunohistochemistry, 200x magnification. The percentage of $CD73^{+}$ immune cells are illustrative examples for each immune phenotype (Immune-Desert, Immune-Excluded, Immune-Inflamed)
