# Supplementary material for: CD39/CD73-mediated immunosuppression and tumor aggressiveness in bladder cancer
Source: Cancer Immunol Immunother. 2026 Apr 22;75(5):154. doi: 10.1007/s00262-026-04400-4 (PMC13103164; doi:10.1007/s00262-026-04400-4)
Supplement: Supplementary file 5 — Supplementary file5 (PDF 393 KB) [file 262_2026_4400_MOESM5_ESM.pdf]

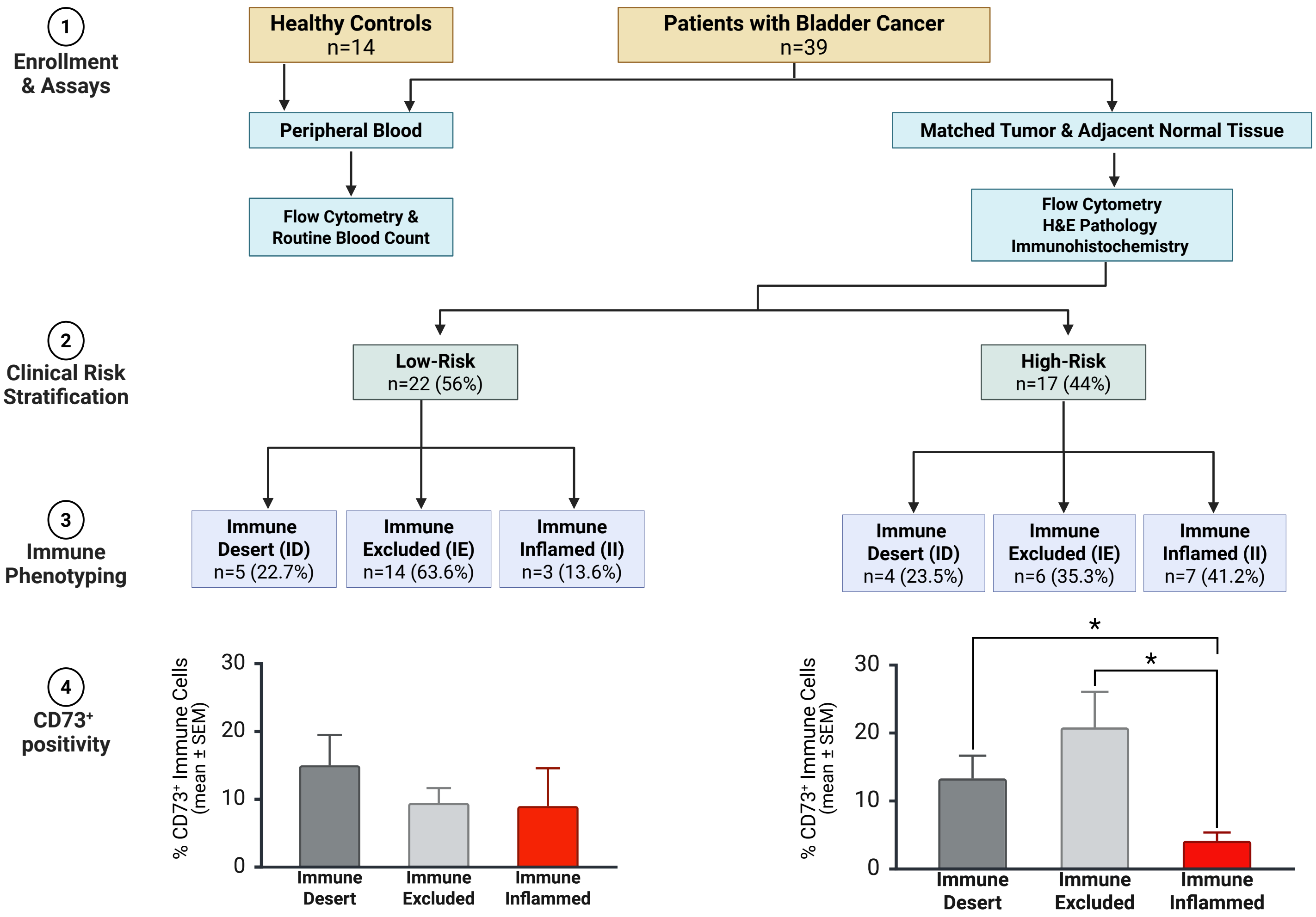

Supplementary Figure 5 Study workflow and clinical-spatial stratification. (1) Patient enrollment (n=39 bladder cancer [BCa], n=14 healthy controls) and matched sample collection for flow cytometry and immunohistochemistry. (2) Stratification into Low-Risk (LR) and High-Risk (HR) groups. (3) Spatial immune phenotyping of the tumor microenvironment into Immune Desert (ID), Immune Excluded (IE), and Immune Inflamed (II) subgroups. (4) Quantification of CD73+ immune cells across spatial phenotypes, demonstrating a significant reduction in II tumors, fundamentally driven by HR cases. * $p<0.05$, when comparing the groups indicated in the figure. Statistical significance was determined using the Mann-Whitney U test. SEM, standard error of the mean
