# Supplementary material for: CD39/CD73-mediated immunosuppression and tumor aggressiveness in bladder cancer
Source: Cancer Immunol Immunother. 2026 Apr 22;75(5):154. doi: 10.1007/s00262-026-04400-4 (PMC13103164; doi:10.1007/s00262-026-04400-4)
Supplement: Supplementary file 6 — Supplementary file6 (DOCX 33 KB) [file 262_2026_4400_MOESM6_ESM.docx]

Supplementary Table 1 - Immune populations in peripheral blood

| **Population** | **Control (n=14)**  **(mean±SD)** | **BC patients (n=39)**  **(mean±SD)** | ***p***  ***(Control vs. BC patients)*** | **Low-risk Patients (n=22)**  **(mean±SD)** | **High-risk Patients (n=17)**  **(mean±SD)** | ***p (low-risk vs. high-risk)*** | ***p (low-risk vs. contro)*** | ***p (high-risk vs. control)*** |
| --- | --- | --- | --- | --- | --- | --- | --- | --- |
| **Routine Blood Count Data** | | | | | | | | |
| Leucocyte count (×10^9^/L) | 7.93±2.02 | 7.97±6.74 | NS | 7.18±1.62 | 9.01±10.12 | NS | NS | NS |
| Populations (% of leucocytes) |  |  |  |  |  |  |  |  |
| Lymphocytes | 25.68±6.85 | 21.27±10.72 | NS | 21.33±11.14 | 21.20±10.44 |  | NS | NS |
| Neutrophils | 53.56±7.43 | 62.47±10.94 | 0.01 | 62.52±10.24 | 62.41±12.04 | NS | 0.01 | 0.043 |
| Monocytes | 7.49±1.90 | 6.65±1.92 | NS | 6.95±1.82 | 6.30±2.04 | NS | NS | NS |
| Eosinophils | 3.39±3.84 | 2.83±2.28 | NS | 0.03±2.51 | 3.00±2.04 | NS | NS | NS |
| Neutrophil-to-Limphocyte Ratio (NLR) | 2.28±0.76 | 4.51±4.46 | 0.036 | 4.84±4.96 | 4.11±3.89 | NS | NS (0.053) | NS (0.08) |
| **Flow Cytometry Data** | | | | | | | | |
| **Lymphocytes** |  |  |  |  |  |  |  |  |
| T cells (CD3^+^) (% of leucocytes)  Subpopulations (%) | 18.9±4.4 | 15.7±8.0 | NS | 15.3±8.6 | 15.8±7.2 | NS | NS | NS |
| CD4^+^ | 63.15±13.13 | 59.44±13.52 | NS | 58.87±11.92 | 60.17±15.71 | NS | NS | NS |
| Phenotypes of CD4^+^ (%)  Th1 (CCR5^+^)  Th2 (CCR4^+^)  Th17 (CCR6^+^)  Subtypes of CCR6^+^ (%)  CCR4^-^/CCR5^-^  CCR4^-^/CCR5^+^  CCR4^+^/CCR5^-^  CCR4^+^/CCR5^+^ | 28.50±12.66  1.82±1.06  25.97±12.80  39.78±8.30  44.81±7.78  3.58±1.85  11.84±4.22 | 17.82±11.48  1.90±1.38  30.52±16.43  51.26±15.33  34.77±15.92  6.04±3.50  7.92±5.15 | 0.002  NS  NS  0.003  0.001  0.015  0.002 | 19.72±11.93  2.19±1.51  30.67±11.63  50.75±17.49  35.87±19.05  6.22±3.76  7.16±2.94 | 15.37±10.73  1.54±1.14  30.32±21.54  51.92±12.49  33.34±11.04  5.82±3.24  8.90±7.05 | NS  NS  NS  NS  NS  NS  NS | 0.017  NS  NS  NS  NS  NS  NS | <0.001  NS  NS  NS  NS  NS  NS |
| CD4^+^ Treg | 5.53±1.31 | 7.73±2.79 | 0.003 | 7.29±2.43 | 8.28±3.19 | NS | 0.023 | 0.001 |
| CD8^+^ | 27.81±11.46 | 35.95±13.33 | 0.048 | 36.25±12.12 | 35.56±15.13 | NS | 0.045 | NS |
| Phenotypes of CD8+ (%)  Tc1 (CCR5^+^)  Tc2 (CCR4^+^)  Tc17 (CCR6^+^)  Subtypes of CCR6+ (%)  CCR4^-^/CCR5^-^  CCR4^-^/CCR5^+^  CCR4^+^/CCR5^-^  CCR4^+^/CCR5^+^ | 67.78±10.15  1.80±5.07  11.08±15.64  13.42±8.85  78.74±13.46  2.33±5.40  5.52±8.07 | 54.65±21.82  1.31±2.35  6.75±15.47  18.05±10.28  73.84±17.88  2.12±2.23  5.99±16.39 | NS  NS  NS  NS  NS  NS  NS | 59.80±19.49  1.60±2.89  3.59±2.74  18.84±10.92  73.01±15.05  2.42±2.43  5.73±14.97 | 47.97±23.41  0.93±1.36  10.85±22.96  17.04±9.60  74.91±21.45  1.73±1.94  6.32±18.55 | NS  NS  NS  NS  NS  NS  NS | NS  NS  NS  NS  NS  NS  NS | NS  NS  NS  NS  NS  NS  NS |
| CD8^+^ Treg | 0.14±0.15 | 0.32±0.32 | 0.033 | 0.30±0.32 | 0.34±0.32 | NS | 0.045 | NS (0.084) |
| CD4^+^CD8^+^ | 0.57±0.55 | 0.80±1.05 | NS | 0.65±0.94 | 1.01±1.17 | NS | NS | NS |
| γδ | 8.52±8.80 | 2.60±2.07 | <0.001 | 3.50±2.44 | 2.54±1.72 | NS | 0.015 | <0.001 |
| CD4^-^CD8^-^γδ^-^ | 0.81±0.40 | 0.70±0.60 | NS | 0.69±0.60 | 0.72±0.63 | NS | NS | NS |
| **Monocytes** |  |  |  |  |  |  |  |  |
| Subtype (%)  Classical (CD14^+^CD16^-^)  Non-classic (CD14^dim^CD16^+^)  Intermediate (CD14^+^CD16^+^)  Phenotype (%)  M1 (CD206^-^)  M2 (CD206^+^) | 83.70±14.32  1.50±1.23  16.30±14.32  99.59±0.95  0.41±0.95 | 86.20±14.15  3.10±2.92  13.80±14.15  99.05±2.30  0.95±2.30 | NS  NS  NS  0.006  NS | 87.62±14.74  3.16±2.88  12.38±14.74  99.38±0.65  0.62±0.65 | 84.52±13.68  3.01±3.07  15.48±13.68  98.67±3.33  1.33±3.33 | NS  NS  NS  NS  NS | NS  NS  NS  0.008  NS | NS  NS  NS  0.022  NS |
| **Natural Killer Cells** (% of leucocytes) | 22.97±13.98 | 21.92±11.06 | NS | 22.66±13.17 | 21.66±6.83 | NS | NS | NS |
| **Dendritic Cells** (% of leucocytes)  CD206^+^ (% of dendritic cells)  Myeloid (CD33^+^) (% of dendritic cells) | 0.11±0.07  5.36±3.77  78.96±14.41 | 0.07±0.07  14.00±10.25  79.96±18.01 | 0.032  0.003  NS | 0.07±0.04  13.36±6.32  78.53±16.32 | 0.08±0.1  14.76±13.70  81.64±20.20 | NS  NS  NS | 0.04  <0.001  NS | NS (0.08)  0.033  NS |

SD, standard deviation; NS, non-statistically significant
