# Supplementary material for: CD39/CD73-mediated immunosuppression and tumor aggressiveness in bladder cancer
Source: Cancer Immunol Immunother. 2026 Apr 22;75(5):154. doi: 10.1007/s00262-026-04400-4 (PMC13103164; doi:10.1007/s00262-026-04400-4)
Supplement: Supplementary file 7 — Supplementary file7 (DOCX 22 KB) [file 262_2026_4400_MOESM7_ESM.docx]

Supplementary Table 2 - Immune populations in tumor and normal tissue

| **Population** | **Normal Tissue**  **(mean±SD)** | **Tumor Tissue**  **All cases**  **(mean±SD)** | ***p***  ***(normal vs tumor tissue, all cases)*** | **Tumor Tissue**  **Low-risk**  **(mean±SD)** | **Tumor Tissue**  **High-risk**  **(mean±SD)** | ***p (low-risk vs. high-risk)*** |
| --- | --- | --- | --- | --- | --- | --- |
| **Lymphocytes** (% of cells) | 13.68±11.00 | 3.63±5.79 | <0.0001 | 3.28±5.92 | 4.12±5.75 | NS |
| T cells (CD3^+^) (% of cells) | 11.07±12.16 | 3.46±5.83 | 0.005 | 3.10±4.18 | 3.96±7.67 | NS |
| Subpopulations (%) |  |  |  |  |  |  |
| CD4^+^ | 32.96±19.02 | 44.13±3.79 | 0.0007 | 41.35±21.78 | 46.86±20.79 | NS |
| Phenotypes of CD4^+^  Th1 (CCR5^+^)  Th2 (CCR4^+^)  Th17 (CCR6^+^)  Subtypes of CCR6+ (%)  CCR4^-^/CCR5^-^  CCR4^-^/CCR5^+^  CCR4^+^/CCR5^-^  CCR4^+^/CCR5^+^  CD4+ Treg | 23.38±18.44  0.80±1.13  40.13±15.42  49.20±25.50  40.66±22.05  4.25±7.77  5.90±9.41  0.63±0.26 | 27.67±20.52  1.39±1.90  22.07±20.88  52.61±27.57  40.57±25.59  2.87±4.19  3.93±5.71  20.29±0.49 | NS  NS  <0.001  NS  NS  NS  NS  0.0008 | 31.51±21.69  1.73±1.91  25.77±19.68  47.70±28.67  43.96±26.37  3.21±5.20  5.13±6.87  2.51±3.21 | 22.40±18.13  0.93±1.86  16.98±22.03  59.37±25.28  35.92±24.54  2.41±2.25  2.30±3.10  2.09±2.62 | NS  0.019  0.019  NS  NS  NS  NS  NS |
| CD8^+^  Phenotypes of CD8+  Th1 (CCR5^+^)  Th2 (CCR4^+^)  Th17 (CCR6^+^)  Subtypes of CCR6+ (%)  CCR4^-^/CCR5^-^  CCR4^-^/CCR5^+^  CCR4^+^/CCR5^-^  CCR4^+^/CCR5^+^ | 62.19±20.16  45.01±28.77  0.01±0.03  9.61±10.17  55.23±37.26  41.81±33.90  0.35±0.82  2.99±8.29 | 46.98±3.95  31.36±26.23  0.13±0.34  7.73±15.43  52.73±32.20  41.49±30.74  0.99±2.37  2.28±6.47 | <0.0001  0.007  0.012  0.03  NS  NS  NS  NS | 49.81±20.31  40.19±28.25  0.15±0.40  7.00±11.60  40.42±30.66  51.26±30.05  0.55±1.46  3.24±8.28 | 39.28±19.51  19.23±17.55  0.10±0.26  8.72±19.92  69.65±26.73  28.04±27.10  1.61±3.19  0.95±1.96 | NS  0.019  NS  NS  0.005  0.021  NS  NS |
| CD4^+^CD8^+^ | 0.71±1.33 | 0.93±0.20 | NS | 1.04±0.92 | 0.79±0.95 | NS |
| γδ | 1.43±1.29 | 1.13±1.96 | NS | 1.50±3.59 | 1.55±2.36 | NS |
| CD4^-^CD8^-^γδ^-^ | 1.36±2.01 | 8.63±14.62 | 0.002 | 6.55±8.14 | 11.49±20.47 | NS |
| **Neutrophils** (% of cells) | 8.31±8.25 | 1.80±3.65 | 0.0002 | 1.98±4.56 | 1.55±1.91 | NS |
| **Macrophages** (% of cells) | 3.25±2.25 | 1.30±2.25 | 0.008 | 1.38±2.30 | 1.18±2.23 |  |
| Subtype (%)  Classic (CD16^-^)  Non-classic (CD16^+^)  Phenotype (%)  M1 (CD206^-^)  M2 (CD206^+^) | 95.62±8.11  4.38±8.12  48.67±29.37  51.33±29.37 | 90.45±2.37  9.55±2.37  42.77±5.28  57.23±5.28 | NS  NS  NS  NS | 93.33±10.78  6.67±10.79  34.60±25.56  65.40±25.56 | 90.63±9.72  9.37±9.73  43.92±27.27  56.08±27.27 | 0.033  0.033  NS  NS |
| **Natural Killer Cells** (% of cells) | 1.13±0.96 | 0.12±0.13 | <0.0001 | 0.09±0.12 | 0.15±0.15 | NS |
| **Non-hematopoietic cells** (% of cells) | 75.09±13.81 | 93.29±9.11 | <0.0001 | 93.35±10.09 | 93.19±7.88 | NS |

*SD, standard deviation; NS, non-statistically significant*
