# Supplementary material for: CD39/CD73-mediated immunosuppression and tumor aggressiveness in bladder cancer
Source: Cancer Immunol Immunother. 2026 Apr 22;75(5):154. doi: 10.1007/s00262-026-04400-4 (PMC13103164; doi:10.1007/s00262-026-04400-4)
Supplement: Supplementary file 8 — Supplementary file8 (DOCX 26 KB) [file 262_2026_4400_MOESM8_ESM.docx]

Supplementary Table 3 - Immunohistochemical Characterization of Tumor Tissue by Risk Group

| **Immunohistochemistry parameter** | **Tumor tissue**  **All cases, n=39**  **(mean±SD)** | **Tumor Tissue**  **Low-risk, n=22**  **(mean±SD)** | **Tumor Tissue**  **High-risk, n=17**  **(mean±SD)** | ***p***  ***(Low-risk vs. High-risk)*** |
| --- | --- | --- | --- | --- |
| **CD4^+^ T cells** |  |  |  |  |
| CD4^+^ Tumor Area (%) | 40.8±27.6 | 34.8±24.6 | 48.2±29.9 | NS |
| Intratumoral Density (cells/HPF) | 14.2±8.1 | 12.3±8.8 | 16.5±6.6 | 0.05 |
| Peritumoral Density (cells/HPF) | 55.1±25.7 | 57.1±25.3 | 52.6±26.7 | NS |
| Staining Intensity (0-3) | 3.0±0.0 | 3.0±0.0 | 3.0±0.0 | NS |
| **CD8^+^ T cells** |  |  |  |  |
| CD8^+^ Tumor Area (%) | 19.5±15.9 | 19.5±17.7 | 19.5±13.8 | NS |
| Intratumoral Density (cells/HPF) | 6.1±8.5 | 6.6±10.9 | 5.5±4.1 | NS |
| Peritumoral Density (cells/HPF) | 24.0±17.8 | 23.1±15.0 | 25.1±21.3 | NS |
| Staining Intensity (0-3) | 3.0±0.0 | 3.0±0.0 | 3.0±0.0 | NS |
| **AP markers on Tumor Cells** |  |  |  |  |
| CD39^+^ Tumor Cells (%) | 35.4±31.9 | 29.8±29.8 | 42.4±34.0 | NS |
| CD39 Staining Intensity on Tumor Cells (0-3) | 1.1±0.7 | 0.9±0.9 | 1.2±0.8 | NS |
| CD73^+^ Tumor Cells (%) | 16.4±19.9 | 23.7±24.1 | 7.4±5.9 | 0.023 |
| CD73 Staining Intensity on Tumor Cells (0-3) | 1.5±1.1 | 1.7±1.1 | 1.2±1.0 | NS |
| A2AR Tumor Cells (%) | 100.0±0.0 | 100.0±0.0 | 100.0±0.0 | NS |
| A2AR Staining Intensity on Tumor Cells (0-3) | 3.0±0.0 | 3.0±0.0 | 3.0±0.0 | NS |
| A2BR Tumor Cells (%) | 73.7±20.1 | 71.0±19.7 | 77.1±20.5 | NS |
| A2BR Staining Intensity on Tumor Cells (0-3) | 1.1±0.2 | 1.0±0.0 | 1.1±0.3 | NS |
| **AP markers on Immune Cells** |  |  |  |  |
| CD39^+^ Immune Cells (%) | 96.6±5.8 | 96.2±6.7 | 97.1±4.7 | NS |
| CD39 Staining Intensity on Immune Cells (0-3) | 2.9±0.3 | 2.9±0.3 | 2.9±0.2 | NS |
| CD73^+^ Immune Cells (%) | 11.2±9.5 | 10.7±8.5 | 11.7±10.9 | NS |
| CD73 Staining Intensity on Immune Cells (0-3) | 1.6±0.8 | 1.6±0.7 | 1.5±0.8 |  |
| A2AR Immune Cells (%) | 93.8±8.7 | 90.5±10.1 | 97.9±3.6 | 0.009 |
| A2AR Staining Intensity on Immune Cells (0-3) | 3.0±0.0 | 3.0±0.0 | 3.0±0.0 |  |
| A2BR Immune Cells (%) | 39.9±20.6 | 33.3±17.9 | 47.9±21.3 | 0.045 |
| A2BR Staining Intensity on Immune Cells (0-3) | 1.5±0.5 | 1.5±0.5 | 1.5±0.5 | NS |
| **PD-1** |  |  |  |  |
| PD-1 tumor cells (%) | 40.3±29.9 | 40.5±28.9 | 40.0±32.0 | NS |
| PD-1 Staining Intensity on Tumor Cells (0-3) | 0.8±0.4 | 0.9±0.4 | 0.8±0.4 | NS |
| PD-1 immune cells (%) | 31.1±15.3 | 30.0±16.4 | 32.4±14.3 | NS |
| PD-1 Staining Intensity on Immune Cells (0-3) | 2.8±0.5 | 2.8±0.5 | 2.9±0.5 | NS |
| **PD-L1** |  |  |  |  |
| ICS | 11.4±12.8 | 11.3±13.3 | 11.6±12.6 | NS |
| CPS | 10.7±14.6 | 11.9±17.3 | 9.3±10.7 | NS |
| TPS | 3.4±8.7 | 3.6±10.9 | 3.1±5.1 | NS |
| **HIF-1α** |  |  |  |  |
| HIF-1α^+^ Tumor Cells (%) | 88.9±22.9 | 83.3±29.4 | 95.9±6.2 | NS |
| HIF-1α Staining Intensity on Tumor Cells (0-3) | 2.2±0.9 | 2.0±0.9 | 2.4±0.7 | NS |
| HIF-1α^+^ Immune Cells (%) | 54.2±21.8 | 49.8±23.1 | 59.7±19.2 | NS |
| HIF-1α Staining Intensity on Immune Cells (0-3) | 1.6±0.6 | 1.6±0.6 | 1.6±0.6 | NS |
| **Immune phenotype,** n (%) |  |  |  |  |
| Immune-desert | 9 (23.1) | 5 (22.7) | 4 (23.5) | NS |
| Immune-excluded | 20 (51.3) | 14 (63.6) | 6 (35.3) | NS |
| Immune-inflamed | 10 (25.6) | 3 (13.6) | 7 (41.2) | NS |

SD, standard deviation; NS, non-statistically significant; ICS, Immune Cell Score; CPS, Combined Positivity Score; TPS, Tumor Proportion Score;
